# Case of Poisoning by Arsenious Acid

**Published:** 1851-03

**Authors:** G. W. Brown

**Affiliations:** Port Carbon, Pa.


### *Case of Poisoning by Arsenious Acid.* By G. W. Brown, M. D., of Port Carbon, Pa.

To the Editors of the Medical Examiner.

Gentlemen,—I enclose you my notes of a case of supposed poisoning by arsenic, which was the subject of a criminal trial at a late term of our court, in Schuylkill county, Pa. Should you deem them of sufficient interest, I should be glad to see them published in the Examiner.

The subject of the poisoning, was a mulatto woman, the wife of a colored man, named Samuel Bertolett. They resided about seven miles from my house, and had always lived peaceably together till within the last two years; since which time Bertolett had become intimate with a colored woman, named Turner, who had left her husband.

Bertolett and his wife had had eleven children, the eldest quite a grown up man. Some time during the summer of 1849, Bertolett took the woman, Turner, home to his house, and gave her complete control of his establishment, not excepting his wife and children. This, of course, gave rise to frequent bickerings and quarrels between Bertolett and his wife, and between the latter and Turner; and thus things went on till the 12th of November, 1849. On that morning, Bertolett, before going to his work, told his little daughter, some 14 years of age, that if her mother should be taken sick during the day, she should come for him. Between eleven and twelve o'clock, Mrs. Bertolett, as was her usual custom, took her husband's dinner to him, a distance of a mile, while Turner remained at home to prepare the dinner for the rest of the family. Immediately on the return of Mrs. Bertolett, she, with the rest of the family, were called to dinner by Turner. The meal consisted of cabbage, potatoes, pork, bread, and coffee. Directly after seating themselves at the table, the little daughter made the observation, that her mother's coffee looked different from the rest. It had the appearance of pepper floating on the top of the liquid. Mrs. Bertolett remarked at the same time, that her coffee had rather a strange taste; but Turner replied that the children had been playing with the pepper box, and she supposed they had spilled some upon the coffee. After dinner, Mrs. Bertolett started to go to a neighboring village, to make some purchases; but, on the way, she was seized with a severe burning pain in the stomach, accompanied by violent retching and vomiting, which continued incessantly, till near eleven o'clock, when she died. About seven o'clock, P. M., Dr. D. J. McKibbin was called in, and found her laboring under the above symptoms, with the addition of cold extremities, inability to speak, clammy perspiration, bloody diarrhœa, and all the symptoms of poisoning from arsenic. But, from the fact of her having partaken of such indigestible food for her dinner, and suspicion not being aroused, he supposed it to be a severe case of cholera morbus, and prescribed accordingly. He staid with his patient till near ten o'clock, P. M., when from the persistence of the symptoms in spite of the remedies, and from a careful review of the case, he told her husband that he believed his wife was laboring under the effects of an irritant poison of some kind, of which she would die in a short time, and she did die in half an hour afterwards.

On Wednesday, 14th November, 1849, I was called to make a post mortem examination of the body; and with the assistance of Drs. McKibbin and Robinson, I proceeded to perform that duty. The body was stretched to the full length, in the usual way, preparatory to interment, with the hands resting upon the abdomen, on either right and left iliac region.

The countenance presented the appearance of extreme suffering; the muscles were nearly as rigid as usual; the ends of the toes and fingers were of a dark leaden hue; the back, sides, and posterior parts of the legs and arms, presented a dark and mottled appearance; the abdomen was slightly tympanitic; under each hand there was a dark green spot; and in the right groin, immediately over one of the absorbent glands, was an old ulcer partially cicatrized. Upon opening the thorax, we found the pleuræ and pericardium pale, but healthy in appearance; the heart very much distended in all its cavities with a dark fluid blood, but otherwise healthy. The lungs, on their anterior portion, were healthy, but rather paler than natural; the posterior portion of them was congested, and of a very dark appearance, looking as though ink had been applied to them. On opening the abdomen, we found the peritoneum in a state of high injection, so much so as to give it a reddish appearance, and upon raising it up to the light, it resembled very much an artificial preparation, in which the arteries had been injected by vermilion. As we approached the stomach and bowels, the redness increased, and continued over these organs. We now tied the œsophagus and duodenum, and removed the stomach, which contained about a pint of fluid and solid matters. We next examined the intestines by tying and laying them open. The mucous surface was covered with a thick, bloody mucus, and was of a deep red color, approaching to brown, with here and there deep yellow spots; and upon examining these spots more closely, we could discover small ulcers in the centre, with softening of the surrounding tissue, so that we could separate it from the muscular coat beneath by very slight pressure with the finger. These yellow spots, containing the softened and ulcerated patches, were spread over the intestines as low down as the ileo-cœcal valve, and were deepest in the centre, and gradually lost their yellowness as the circumference was approached, till they were merged in the surrounding redness.

The liver was gorged with blood, as was also the spleen, but they were otherwise healthy. The bladder was small and contracted, and its coats appeared thickened. The uterus appeared healthy, as were also its appendages, and the external genitals. After removing the stomach, and placing it in a covered vessel, I

took it home with me, when we proceeded to examine it. The contents were emptied into a glass vessel and tightly corked, and the stomach laid open along its lesser curvature. The mucous membrane was covered with the same kind of bloody mucus as the intestines; but we could not detect any shining particles in it. It presented the same appearance as that of the intestines, but was of a much deeper red, and the yellow patches were more numerous and larger. At the large end of the stomach there was one as large as a Spanish dollar, with two large ulcers in the centre, and the mucous membrane was softened so as to be easily separated from the muscular coat beneath. There were several large patches near the pyloric orifice of the stomach, also containing ulcers.

The mucous membrane of the œsophagus presented a red appearance, but was not nearly so dark as that of either the stomach or intestines. The stomach was now cut up into small pieces, and put into the glass vessel with the contents; some water and acetic acid were added, and the vessel was placed in a water bath, and boiled until I was satisfied that any arsenic it might contain must be dissolved. I then strained the liquid through a coarse cloth, so as to separate the liquid from the solid matters, and then filtered through paper, allowing the solution to stand for a day or two, when I afterwards filtered again. I now took an eight-ounce vial, and put into it eight grains of arsenious acid, and eight grains of the carbonate of potash, and filled the vial with water, placing the whole in a water bath till complete solution was effected. I now took two two-ounce vials, and placed an ounce of the solution of the stomach into the one, and an ounce of the solution of the arsenite of potash into the other, and then added sufficient acetic acid to both to neutralize any alkali they contained, or coagulate any organic matters. I then added to each vial a portion of a solution of the ammonio-sulphate of copper, when, immediately, a deep green precipitate was thrown down to the bottom of each, so nearly resembling each other, that scarcely a shade of difference was perceptible. I then applied the ammonio-nitrate of silver in the same way, but produced no precipitate whatever. This experiment I repeated several times, with the same results. I now placed about eight ounces of the solution of the contents of the stomach in a proper apparatus, provided for the purpose, and added acetic acid as before, and passed through it a stream of sulphuretted hydrogen gas, when immediately a deep lemon yellow precipitate was formed on the top of the liquid, and gradually fell, in flocculent masses, to the bottom of the vessel. After letting it stand for some time, I removed it and washed out the vessel, and placed in it the same quantity of the solution of the arsenite of potash, and passed through it the sulphuretted hydrogen gas, when the same kind of precipitate was formed on the top of the liquid, and fell to the bottom of the vessel in the same way. I now collected the sulphuret of arsenic precipitated from the solution of the stomach, by filtering and drying on a watch glass, and placed it in a tube with the black flux, and attempted to sublime it. After having, however, held the bulb in the flame of a spirit lamp for some time, when light fumes began to escape from the mouth of the tube, and a white crystalline powder collected upon the sides of the tube, (where it was kept cool by a pair of large iron pincers, by which I held it,) the tube began to get red; and before the metal was reduced, it burst, and left a hard grey mass, resembling the slag of a furnace, at the bottom. It had not the metallic lustre, however. The white crystalline powder I supposed to be the arsenious acid.

The opinion given by myself and the other medical witness was, that from the symptoms preceding death, from the post mortem appearances after death, and from the chemical tests, the patient came to her death by poisoning from arsenic. The yellow spots on the mucous membrane of the stomach and bowels we supposed were produced by a chemical action having taken place between the arsenic and the sulphuretted hydrogen gas that always exists in a free state in the alimentary canal, thereby forming the sulphuret of arsenic, and giving them the peculiar yellow color. We also contended that the ulceration and softening of the mucous coat of the stomach and intestines must have been the result of a corrosive poison, as the time from her first symptoms of sickness to her death, was too short for it to be the result of inflammation. Were we justified in giving such an opinion? Or, in other words, was the evidence such as to bear us out in such a conclusion? I would respectfully solicit the opinion of any of the readers of the Examiner who may be conversant with the subject, on this important case, as the counsel for the defence dwelt strongly upon the insufficiency of the medical testimony. The prisoner was cleared, although it was clearly proven that he had bought arsenic, and had it in the house at the time of his wife's death ; that he had been, and was still, living in criminal intercourse with the woman, Turner ; that he had frequently threatened he would kill his wife, and that Turner was pregnant by him at the time of his wife's death, and was confined some two months after she was placed in prison. The principal chemical authority, quoted by the counsel, was Cooper's, a work on that subject written some forty years since. The test of the solution of the stomach by sulphuretted hydrogen, exhibited before the Court, has since been confirmed by *Marsh's* apparatus, and the metallic ring formed.

I am, gentlemen, very respectfully,

Your obedient servant,

G. W. BROWN.

P. S. It is more than probable that nux vomica was administered first in the coffee at dinner, and, as it did not produce the desired effect soon enough, the man gave his wife arsenic after he came home. Nux vomica was found in large quantities in the house afterwards, and its presence will account for the peculiar appearance on the coffee at dinner.

*Port Carbon, January,* 1851.

[From the facts submitted by our correspondent, and in the absence of opposing testimony, we have no hesitation in expressing the opinion that a verdict *against* the prisoner, Bertolett, was called for. The woman was undoubtedly poisoned by arsenious acid, administered by her husband, either directly, or through the agency of Turner.—EDS. EXAMINER.]

